# Relationship between HSP90a, NPC2 and L-PGDS proteins to boar semen freezability

**DOI:** 10.1186/s40104-017-0151-y

**Published:** 2017-03-01

**Authors:** Julián Valencia, Germán Gómez, Walter López, Henry Mesa, Francisco Javier Henao

**Affiliations:** Universidad de Caldas, Faculty of Agricultural Sciences, A.A. 275, Manizales, Caldas Colombia

**Keywords:** Boar freezability, HSP90a, L-PGDS, NPC2, Semen preservation, Seminal plasma

## Abstract

**Background:**

The purpose of this study was to determine the association of three proteins involved in sperm function on the freezability of porcine semen: the heat shock protein 90 alpha (HSP90a), the Niemann-Pick disease type C2 protein (NPC2), and lipocalin-type prostaglandin D synthase (L-PGDS).

Six adult boars (each boar was ejaculated three times, 18 in total) were classified by freezability based on the percentage of functionally competent sperm. The male semen with highest freezability (MHF) and the male semen with lowest freezability (MLF) were centrifuged immediately after collection to separate seminal plasma and spermatozoa to make four possible combinations of these two components and to incubate them for 3 h, adjusting the temperature to 17 °C, to freeze them afterwards. The quantification of proteins was performed in two stages: at zero and at 3 h after incubation of the four combinations.

**Results:**

The spermatozoa × incubation time (IT) interaction only had effect (*P* < 0.01) on HSP90a levels; this protein increased in seminal plasma, after 3 h of incubation, in larger quantity (*P* < 0.05) in combinations with MLF spermatozoa. In relation with the NPC2 protein, two isoforms of 16 and 19 kDa were identified. The 19 kDa isoform was affected (*P* < 0.01) only by the seminal plasma × IT interaction, with superior values (*P* < 0.01) both at zero and three hours of incubation, in the combinations with MHF seminal plasma; and 16 kDa isoform was affected (*P* < 0.01) only by the IT with reduction after 3 h of incubation. The levels of L-PGDS was affected (*P* < 0.01) only by the spermatozoa × IT interaction, which reduced (*P* < 0.01) in combinations with MLF spermatozoa after 3 h of incubation.

**Conclusions:**

It is possible to consider that the three proteins evaluated were associated with freezability of boar semen due, especially, to the fact that mixtures with MLF spermatozoa showed greater increase levels of the HSP90a protein and reduction of L-PGDS in plasma. In addition, the seminal plasma of MHF had higher concentration of the NPC2 of 19 kDa protein, which was reduced by incubating with MHF spermatozoa.

## Background

The quality of frozen-thawed boar semen shows strong variability associated with differences in freezability between individuals [1], apparently determined by both seminal plasma and sperm characteristics [2]. Some studies have reported significant associations between several seminal proteins and freezability of boar semen, some located in the seminal plasma such as fibronectin type 1 [3] and β subunit of N-acetyl-β-hexosaminidase [4], and others in the sperm cell, such as the heat shock protein 90 alpha [5], the acrosin binding protein and the triosephosphate isomerase protein [6].

From the work done by Pursel et al. [7] it was suggested that incubating semen for a period of 3 to 5 h at temperatures above 15 °C increases the resistance of spermatozoa to cold shock. With this same approach, Casas and Althouse [8] found greater stability and integrity of the sperm membrane after the thermal shock at 5 °C, and Yeste et al. [9] demonstrated greater sperm resistance to freezing-thawing processes when semen was previously maintained at 17 °C. This period has been called “holding time”. These findings and the aforementioned proteins allow formulating conjectures about biological phenomena that determine the differences in freezability between individuals, which could occur between semen collection and holding time at 17 °C thanks to the interaction between seminal plasma and sperm.

Among the most important effects of freezing on semen quality that stand out is the reduceded sperm survival, and membrane changes and loss of its selective permeability to ions such as calcium, because of a phenomenon called cryocapacitation, which has events that are similar, but no identical, to true sperm capacitation [10]. True sperm capacitation is an event characterized by reordering of the sperm membrane, altered motility pattern, and changes in metabolic activity which allow the acrosome reaction and finally the penetration of the oocyte [11]. Other changes in true capacitation process such as the release of seminal plasma proteins from the surface of the spermatozoa, the loss of membrane cholesterol and signaling induced by reactive oxygen species (ROS), are induced in a disordered manner during freezing [12]. In this respect, there is evidence that ROS can induce sperm capacitation, in a balanced and controlled process, by the activation of adenylate cyclase and cyclic adenosine monophosphate (cAMP); however, freezing produces an excessive production of ROS, which generate lipid peroxidation of membrane fatty acids, DNA damage and macromolecules damage such as proteins and lipids [13].

As a result of cryopreservation, plasma membrane destabilizes and becomes more permeable to Ca^2+^ influx [14]. As a consequence the levels of cAMP [11] and the kinase-32 protein activity increase, resulting in phosphorylation on tyrosine residues of certain proteins among which it is important to mention the binding to proacrosin [15]. In the movement of cholesterol from the plasma membrane, specific molecules of high affinity intervene with it such as the Niemann-Pick type C2 protein which is closely related to the lipid structure of the plasma membrane [16, 17]. It has been confirmed that this protein is the most secreted into the epididymis [17], and it is also secreted into seminal vesicles, prostate and vas deferens [18]. NPC2 protein is considered a decapacitating factor [19] which binds more efficiently those spermatozoa with higher content of cholesterol [18].

Heat shock proteins (HSP) are also involved in the sperm resilience to withstand cryopreservation, possibly as molecular chaperones of starting proteins of the phosphorylation cascades represented by threonine/serine kinases or tyrosine kinase [20]. The above approach is based on the fact that the primary function of HSP is the conservation of these enzymes by correcting defects occurring during folding or damage by denaturation due to thermal or oxidative stress, thus preventing their aggregation in an irreversible way [21]. Among the HSPs, the most studied in boar semen is the HSP90a, which is found intracellularly in lower quantities in low freezability than in higher freezability spermatozoa [5, 22], possibly due to their exit from the spermatozoa into the extracellular space because of the loss in the integrity of the plasma membrane during the cooling [23]. The association between HSP90a and freezability can be attributed to its participation in capacitation and apoptosis processes [5, 22]. Considering that freezing induces apoptosis of sperm reducing the time of viability of these cells [24].

Prostaglandin D synthase lipocalin type (L-PGDS) is an associate protein in boars and bulls with the acrosome reaction [25], with the transformation of prostaglandin H_2_ in prostaglandin D2 [26] and with the transport of lipophilic substances such as retinoid, with high affinity with retinoic acid and retinol [27]. These two latter molecules are involved in the early capacitation [14], because they change the permeability of the plasma membrane [28]. Additionally, L-PGDS is present in the acrosomal membrane of freshly ejaculated spermatozoa and then disappears within the sperm acrosome reaction [25], and the in vitro incubation of sperm with this protein increases their binding capacity to the zona pellucida [29].

The purpose of this study was to determine the dynamics of HSP90a, the NPC2 protein and L-PGDS in seminal plasma during the period between collection and cooling to 17 °C and their effect on freezability of boar semen.

## Methods

### Animals

Six boars between 18 and 43-month-old, five Pietrain and one Landrace × Large White of an insemination center in the central-western Colombia, with normal semen analysis, optimal sanitary and fertility conditions were used for the study. They were kept in individual 2.6 m × 2.5 m pens, fed 2 kg/animal/d of a commercial feed for boars, water at will, and ejaculated every 8 d between 0700 and 0900 h.

### Semen collection and freezing

The six boars were ejaculated three consecutive times (18 ejaculates in total) using the gloved hand technique. The rich fraction of each male was separated visually, diluted 1:1.5 with Androhep® plus (Minitüb, Germany) and transported to the Laboratory of Biology Reproduction at Universidad de Caldas for freezing and seminal evaluation. The transport was performed at a temperature greater than equal to 17 °C.

The freezing protocol used was Minitüb®, developed as follows:

The sperm concentration was determinate by photometry, and an aliquot at 30×10^6^ spermatozoa/mL was prepared for semen analysis. The remaining semen was centrifuged at 17 °C at 800 × g for 20 min, then the pellet was adjusted at a concentration of 2×10^9^ spermatozoa/mL with cooling diluent (Cryo Androstar plus® + 20% egg yolk) at 17 °C, and spermatozoa were cooled down to 5 °C at 1 °C every 7.5 min. Next, spermatozoa were diluted at 1×10^9^ spermatozoa/mL with freezing extender (Cryo Androstar plus® + 20% egg yolk + 6% glycerol) at 5 °C. Spermatozoa were finally packed in 0.5 mL french plastic straws, in a controlled environment at 5 °C. The straws were frozen by exposure to liquid nitrogen vapors (5 cm above the level of nitrogen) for 20 min, and stored into liquid nitrogen. The semen was thawed at 38 °C for 20 s in a water bath, and diluted in Androhep® plus at 26 °C at a rate of 7.5 mL of diluent per straw.

### Evaluation of sperm variables

Sperm variables evaluated were: sperm motility by computer assisted semen analysis (CASA) and software Sperm Class Analyzer (Ver. 5.4, 2013; Microptic S.L., Barcelona, Spain), membrane structural integrity (MSI) by staining with SYBR-14 and propidium iodide (LIVE/DEAD® Sperm Viability Kit L-7011, Molecular Probes, Europe), incubation for 5 min at 37 °C in a water bath, and visualization of 100 spermatozoa at 1,000× magnification in a epifluorescence microscope (Nikon Eclipse 80i, Japan) with BV-2A filter [30], membrane functional integrity (MFI) through the short hypoosmotic swelling test (sHOST) and examination of 100 spermatozoa at 400× magnification in a phase-contrast microscope (Nikon Eclipse 50i, Japan) [31], acrosome integrity in sperm fixed with 2% glutaraldehyde, without staining, and examination of 100 spermatozoa at 400× magnification in a phase-contrast microscope (Nikon Eclipse 50i, Japan) [32] and sperm morphology by counting 100 spermatozoa in semen fixed with formalin saline solution. without staining, at 400× magnification in a phase-contrast microscope (Nikon Eclipse 50i, Japan) [32]. Furthermore, to add credibility to the evaluation [33] an sHOST combined with a viability test [34] was performed as follows: two vials were prepared, vial 1 with 1.5 mL semen at 17 °C and vial 2 with 1 mL of hypoosmotic solution at 75 mOsm/kg, 5 μL of SYBR-14 and 5 μL of PI which were progressively incubated in a water bath at 37 °C; then, 500 μL of semen from vial 1 were transferred to vial 2, which was incubated for 5 min at 37 °C and fixed with 10 μL of 2% glutaraldehyde in BTS to evaluate acrosome domain [32]. Visualization was performed by the counting of 100 spermatozoa in a differential interference contrast and epifluorescence microscope (Nikon Eclipse 80i, Japan) with BV-2A filter at 1,000× magnification. The result of the test was: percentage of functionally competent sperm (PFCS) and percentage of cryocapacitated sperm (CS). PFCS or sperm having the major prerequisites for the penetration through the oocyte [35], except for motility; that is: the total sperm alive, morphologically normal, with membrane functional integrity or sHOST positive, and with acrosome integrity, after membranes stress which emulates part of the effects of freezing. The CS were those sperm sHOST positive alive and with true acrosome reaction [34]. Both populations were adjusted for previous agglutination and coiled tail values. The sperm not functionally competent were those having one or more of the following characteristics: morphological abnormalities, loss of membrane functional integrity or sperm sHOST negative, loss of acrosome integrity, agglutination and cell death.

### Determination of freezability

Three ejaculates from each of the six males once subjected to freezing and thawing processes were evaluated based on PFCS to determine freezability of males. The highest freezability semen is that with highest PFCS after freezing-thawing and the lowest freezability semen is that with lowest PFCS after freezing-thawing.

### Combinations of seminal plasma and sperm with different freezability

The rich fraction of the male with the highest freezability (MHF) and the male with the lowest freezability (MLF), were collected and centrifuged at 800 × g for 10 min at room temperature, immediately after collection to separate the seminal plasma and spermatozoa (SPZ). The seminal plasma obtained was filtered through a 0.2 μm membrane pore size to remove non- separated spermatozoa. With the SPZ and the seminal plasma obtained, four combinations were made, mixing one part spermatozoa with six plasma: Low freezability spermatozoa + High freezability seminal plasma (LFS + HFSP), High freezability spermatozoa + Low freezability seminal plasma (HFS + LFSP), Low freezability spermatozoa + Low freezability seminal plasma (LFS + LFSP) and High freezability spermatozoa + High freezability seminal plasma (HFS + HFSP). These mixtures were added with Androhep® (1:1.5) for a concentration of 213 ×10^6^ spermatozoa/mL each combination, and incubated for 3 h adjusting the temperature to 17 °C to be frozen later. The analysis of sperm variables was performed before and after freezing, in frozen semen stored for 15 d.

### Identification and quantification of proteins in seminal plasma

The seminal plasma intended for quantification of proteins was obtained in two stages: on the farm immediately after the collection (0 h) by centrifugation at 800 × g for 10 min a room temperature, and in the laboratory after 3 h of incubation. Both seminal plasma samples were stored at −20 °C.

The total protein concentration estimated by the Bradford method (BIO-RAD Laboratories, Inc., Hercules, CA) at 595 nm wavelength in a UV-visible spectrophotometer (Perkin Elmer Lambda XLS, UK). Protein separation was carried out by sodium dodecyl sulfate polyacrylamide gel electrophoresis (SDS-PAGE) discontinuous (7% for stacking and 16% for resolution) as follows: 1) Incubation at 95 °C for 5 min, of a 1: 3 dilution of seminal plasma and Laemmli buffer denaturing solution; 2) calculation and sowing of 10 μg total protein per well; and 3) gel run with buffer TRIS-Glycine 50 V for 20 min and then at 100 V for 4 h. The transfer was carried out at constant current of 25 mA for 12 h, to polyvinylidene difluoride (PVDF) membranes previously treated with absolute methanol.

Incubation with the antibodies was conducted as follows: 1) washing of the membrane in Tween 20 at 0.1% in phosphate buffered saline (PBST) for 10 min and with phosphate buffered saline (PBS) for 10 min (twice); 2) blocking with 3% bovine serum albumin (BSA) in PBST for 12 h at 4 °C; 3) washing and addition of primary antibodies (produced in rabbits) diluted in 1% BSA solution in PBST as follows: anti-HSP90a at 1:10,000 dilution, anti-L-PGDS at 1:10,000 dilution and anti-NPC2 at 1:100 dilution, to incubate for 12 h at 4 °C; 4) washing and addition of the secondary antibody conjugated with horseradish peroxidase enzyme (produced in goat, anti-rabbit) at 1:5,000 dilution to hatch for 2 h at room temperature 20 to 22 °C. The development was conducted as recommended by the Opti-4CN Kit (BIO-RAD Laboratories, Inc., USA), and the identification and quantification of the bands obtained was conducted with the ImageLab-4 BIO-RAD software based on density and volume, using Gel Doc^TM^ XR+ (BIO-RAD Laboratories, Inc., Hercules, CA). BSA was used as internal concentration control and the bands were normalized through a measurement of volume and density after staining with Ponceau red prior to incubation with the antibodies, with the specifications for normalization of the ImageLab-4 BIO-RAD software.

### Statistical analysis

All statistical analyses were performed using SAS (SAS Inst. Cary, NC). All sperm quality and protein variables were tested for normality using the Shapiro-Wilk test. Homogeneity of variance for each effect included in the analyses was tested with the Levene (group means) and the Brown and Forsythe (group medians) tests. Variables with normal distribution were analyzed using the GLM procedure, while variables with non-normal distributions with the GENMOD procedure. Correlation analysis were performed with the CORR procedure.

The association between levels of heat shock protein 90 alpha, the Niemann-Pick disease protein type C2 and lipocalin type prostaglandin D synthase, with freezability based on PFCS was analyzed as a factorial arrangement 2×2×2 (seminal plasma and SPZ high and low freezability, and zero and three hours of incubation) with three replicates in a completely randomized design. Variance analysis and multiple comparisons were adjusted using the Tukey’s test. Spearman correlation analysis was carried out among protein concentrations and variables of post-freezing seminal quality. Other seminal variables were analyzed using a factorial 2×2 design (high and low freezability seminal plasma and SPZ), with three replicates in a completely randomized design. The effects of seminal plasma and SPZ, and their interaction were assessed in a log-linear model, consisting of a Poisson regression with a logarithmic function. The results were expressed as least square mean and standard error.

## Results and discussion

### Evaluation of males by freezability

The difference in the freezability of the six boars evaluated based on the PFCS was found in this work (Table 1). Freezability level is a condition that is maintained throughout the productive life of boars [1] allowing their characterization with the evaluation of one or a few ejaculates [36]. The findings of this study are consistent with the above, where the evaluation of three ejaculates per male with an 8 d of interval, allowed identifying males with high and low freezability as seen in Table 1, where is shown that boar 1 (male with the highest freezability), in the three evaluations, presented PFCS higher than boar 6 (male with the lowest freezability).

**Table 1:** PFCS in frozen-thawed semen of six boars in an insemination center of central-western Colombia

Boars	Evaluation 1	Evaluation 2	Evaluation 3	Average
1	5.00	6.72	5.60	5.77
2	6.79	4.44	6.10	5.77
3	4.40	5.34	2.84	4.19
4	3.52	4.20	4.10	3.94
5	3.48	3.12	2.72	3.10
6	1.74	2.70	2.73	2.39

### Relationship of the dynamics of the three plasma proteins with freezability

The interaction of three factors seminal plasma, SPZ and IT, and seminal plasma × SPZ, did not affect (*P* > 0.05) the concentration of the proteins studied. The interaction SPZ × IT was highly significant (*P* < 0.01) on the HSP90a levels, a significant effect (*P* < 0.05) on the levels of the L-PGDS, and had no effect (*P* > 0.05) on the concentration of the two isoforms of NPC2. The interaction seminal plasma × IT only affected (*P* < 0.01) the NPC2 levels of 19 kDa. The seminal plasma factor did not affect the HSP90a and L-PGDS levels, and the SPZ factor did not affect the NPC2 of 19 kDa (*P* > 0.05). The NPC2 16 kDa was affected (*P* < 0.01) only by IT, with a reduction after 3 h of incubation.

### Heat shock protein alpha (HSP90a)

**Fig. 1:**
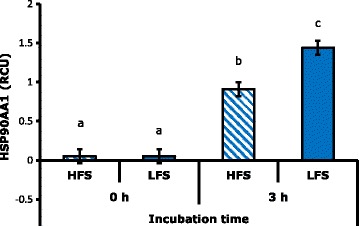
Effect of interaction spermatozoa per incubation time on the concentration of HSP90a in seminal plasma. RCU: relative concentration units. Different letters indicate highly significant differences (*P* < 0.01)

**Fig. 2:**
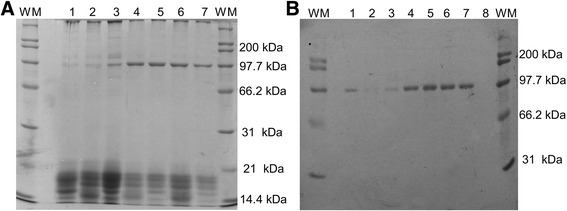
A, Polyacrylamide gel. B, PVDF membrane with anti-HST90a antibody. WM: weight marker, 1: positive control, 2: seminal plasma of MHF, 3: seminal plasma of MLF, 4: HFS + HFSP, 5: LFS + HFSP, 6: HFS + LFSP, 7: LFS + LFSP, 8: negative control with BSA

SPZ × IT interaction had a highly significant effect (*P* < 0.01) on the HSP90a levels, while the seminal plasma factor was not significant (*P* > 0.05). Tukey’s multiple comparisons showed that the concentration of HSP90a on seminal plasma was higher after 3 h of incubation in combinations with SPZ of MLF (Figs. 1 and  2). This protein has multiple cellular functions such as: protection to toxic agents, heat stress [21] and oxidative stress during freezing [22]; modulation of apoptosis [37]; and participation in motility [23] and in sperm maturation [22]. Casas et al. [5] associated high intracellular concentration of this molecule prior to freezing with high freezability males, and Huang et al. [23] mention the possibility of leaking of this protein from spermatozoa because of reduction of the membrane integrity which could explain the results found in this work.

The concentration of the protein HSP90a at 0 h of incubation had a positive correlation not significant with structural membrane integrity (0.51; *P* = 0.08) (Table 2). The concentration of the protein HSP90a after 3 h of incubation had negative and significant correlation with progressive motility (−0.64; *P* < 0.05), negative and not significant correlation with total motility (–0.54; *P* = 0.06) (Table 2). In this regard, Huang et al. [23] found that the decrease of HSP90a in the sperm is associated with low post-freezing motility and Casas et al. [5] showed that high intracellular concentration of this protein pre-freezing has a positive correlation with post-freezing motility.

**Table 2:** Spearman correlation coefficients between three seminal proteins with any post-freezing sperm variables (correlation coefficients/probability value)

Time of incubation	Proteins	Membrane structural integrity	Membrane functional integrity	Acrosome integrity	Progressive motility	Total motility	Percentage of functionally competent sperm
0 h	HSP90a	0.51 0.08	0.21 0.50	–0.15 0.62	0.28 0.36	0.22 0.48	0.08 0.79
	NPC2 19 kDa	–0.44 0.15	0.02 0.93	0.27 0.38	–0.11 0.72	–0.01 0.96	0.20 0.52
	NPC2 16 kDa	–0.50 0.09	0.09 0.75	0.04 0.89	–0.24 0.45	0.02 0.93	0.21 0.50
	L-PGDS	–0.10 0.73	0.27 0.37	–0.13 0.68	–0.22 0.48	–0.20 0.51	–0.10 0.95
3 h	HSP90a	–0.33 0.29	0.34 0.26	–0.47 0.12	–0.64 0.02	–0.54 0.06	–0.21 0.50
	NPC2 19 kDa	–0.16 0.59	0.05 0.86	0.01 0.96	–0.10 0.74	–0.12 0.69	–0.10 0.75
	NPC2 16 kDa	–0.14 0.66	0.39 0.19	–0.18 0.55	–0.26 0.40	–0.07 0.81	0.15 0.64
	L–PGDS	–0.05 0.87	–0.42 0.16	0.43 0.15	0.04 0.89	0.29 0.34	0.31 0.32

*HSP90a* Heat shock protein 90 alpha, *NPC2* Niemann-Pick disease type C2 protein, *L-PGDS* Lipocalin-type prostaglandin D synthase

### Niemann disease protein type C2 protein (NPC2)

**Fig. 3:**
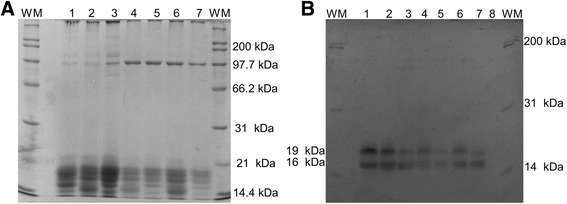
A, Polyacrylamide gel. B, PVDF membrane with anti-NPC2 antibody. WM: weight marker, 1: Positive control, 2: seminal plasma of MHF, 3: seminal plasma of MLF, 4: HFS + HFSP, 5: LFS + HFSP, 6: HFS + LFSP, 7: LFS + LFSP, 8: negative control with BSA

After performing the Western blot of NPC2 protein two isoforms were visualized: 16 and 19 kDa (Fig. 3b) which coincide with that reported by Okamura et al. [17] who found these two isoforms when using polyclonal antibodies to the aforementioned protein.

**Fig. 4:**
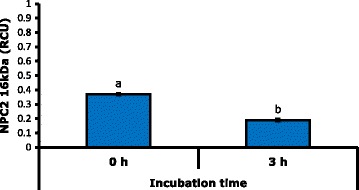
Effect of incubation time on the concentration of NPC2 of 16 kDa. RCU: relative concentration units. Different letters indicate highly significant differences (*P* < 0.01)

**Fig. 5:**
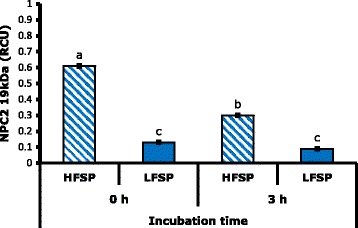
Effect of seminal plasma interaction per incubation time on the concentration NPC2of 19 kDa. RCU: relative concentration units. Different letters indicate highly significant differences (*P* < 0.01)

The interaction IT × seminal plasma was highly significant (*P* < 0.01) on the NPC2 levels of 19 kDa, while the SPZ factor was not significant (*P* > 0.05), and NPC2 16 kDa was affected (*P* < 0.01) only by the IT with a reduction after 3 h of incubation (Fig. 4). Multiple comparisons by Tukey allowed to establish differences in concentration of the NPC2 of 19 kDa isoform by effect of the interaction seminal plasma × IT, which presented a higher concentration in the combinations with seminal plasma of MHF both at zero and 3 h of incubation (*P* < 0.01) with a lower concentration at the end of incubation (Fig. 5). In this regard, it is considered that NPC2 is one of the proteins with higher affinity to cholesterol [16] and high levels of this protein in seminal plasma are related to high cholesterol ratio in the sperm membrane [18]. In this way, it is possible that reduction of NPC2 in seminal plasma after 3 h of incubation is due to union of this protein to sperm membrane.

In this work it was shown that high freezability males have a higher level of NPC2 of 19 kDa in the plasma, which can be associated with a higher level of cholesterol in the sperm membrane and with greater efficiency in the protein binding to spermatic cholesterol. Therefore, this type of male might have a better mechanism of prevention of cryocapacitation. This is based in the fact that the release of cholesterol from the membrane is a factor that triggers the cryocapacitation process and the procedures performed to the semen during freezing can promote this release [38]. Furthermore, NPC2 of 19 kDa, in mixtures with seminal plasma of MLF showed no reduction in the concentration at 3 h of incubation (Fig. 5) which can be related to the loss of its function by oxidation due to the effect of reactive oxygen species in this type of plasma [39].

### Lipocalin type Prostaglandin D synthase (L-PGDS)

**Fig. 6:**
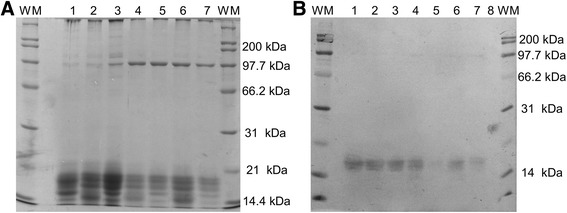
A, Polyacrylamide gel. B, PVDF membrane with anti-L-PGDS antibody. WM: weight marker, 1: positive control, 2: seminal plasma of MHF, 3: seminal plasma of MLF, 4: HFS + HFSP, 5: LFS + HFSP, 6: HFS + LFSP, 7: LFS + LFSP, 8: negative control with BSA

**Fig. 7:**
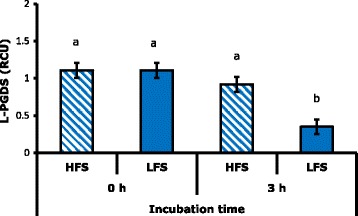
Effect of spermatozoa interaction times incubation time on L-PGDS concentration. RCU: relative concentration units. Different letters indicate highly significant differences (*P* < 0.01)

SPZ × IT interaction was significant (*P* < 0.05) on the levels of this protein, while the seminal plasma factor was not significant (*P* < 0.05). Multiple comparisons by Tukey showed that the concentration of L-PGDS was lower (*P* < 0.01) in SPZ of MLF after 3 h of incubation (Figs. 6 and 7). It is considered that this protein is a carrier of retinoid such as retinoic acid and retinol [27] which affect the permeability of the plasma membrane while interacting with phospholipids, resulting in greater input of ions from the outside [28]. This phenomenon may be related to cryocapacitation due to access of ions such as Ca^2+^ which turns out to be acrosome and hypermotility reaction [14]. Additionally, in vitro incubation of sperm with L-PGDS protein increases its binding to the zona pellucida [29] an action that is possible only after changes in membrane that occur during the capacitation process [38].

It is possible to consider that the three evaluated proteins were associated with freezability of boar semen due, especially, to the fact that mixtures with spermatozoa of the low freezability males recorded higher increase in levels of HSP90a protein, and reduction of L-PGDS in plasma once the incubation period was completed. While combinations with spermatozoa from the high freezability males showed lower HSP90a reduction and no change in the concentration of L-PGDS in the same period, which can ensure greater protection to thermal and oxidative stress by HSP90a [5] and smaller amounts of retinoic acid and retinol that promote changes in the membrane permeability [28]. In addition, the seminal plasma of males of high freezability had greater concentration of the NPC2 protein of 19 kDa which was reduced by incubating with high freezability spermatozoa, which in turn could be associated with a better prevention mechanism of cryocapacitation of these sperm by effect of plasma [38]. However, it is important to mention that the correlation analysis, the concentration of L-PGDS, NPC2 kDa of 19 kDa, and NPC2 of 16 kDa proteins at zero and three hours of incubation had no significant correlation (*P* > 0.05 ) with any post-freezing sperm variable (Table 2). There are evidences that the incubation of the semen at 17 °C for 24 h minimizes the alterations in the membrane fluidity and architecture, and generate resistance of the spermatozoa to cold shock, possibly related to the effect of seminal plasma proteins [8]. Although, in the present study, the incubation in this period was only of 3 h, were found changes in the concentration of the three proteins studied, related to the origin of seminal plasma and spermatozoa (both coming of male with highest or lowest freezability). Although these results provide relevant information on the mechanism by which the holding time reduces the effects of cold shock, it is important to conduct studies with longer incubation periods, including evaluation of the lipid architecture and membrane fluidity, and the effect of seminal plasma proteins in preventing the cholesterol efflux by measuring it in the membrane.

### Effect of sperm and seminal plasma on semen quality

In the descriptive analysis of frozen-thawed seminal quality variables from the four combinations, it was found that acrosome integrity, progressive motility, total motility and PFCS mean values, were highest in the combinations that had high freezability spermatozoa; and the number of cryocapacitated spermatozoa and membrane functional integrity mean values were lowest in these same combinations (Table 3).

**Table 3:** Frozen- thawed seminal quality variables from the four combinations of sperm and seminal plasma of males with high and low freezability

Combinations	Replicates	Membrane structural integrity	Membrane functional integrity	Acrosome integrity	Progressive motility	Total motility	Percentage of functionally competent sperm	Cryocapacitated sperm
HFS + HFSP	1	51.00	38.07	28.00	18.65	36.30	3.24	14.58
	2	47.00	33.75	41.00	20.15	39.07	3.75	6.00
	3	48.00	40.32	26.00	12.88	29.16	3.80	7.60
	Average	48.67	37.38	31.67	17.23	34.84	3.60	9.39
LFS + HFSP	1	54.00	47.84	16.00	11.54	23.08	2.76	23.00
	2	41.00	56.40	12.00	6.14	15.48	2.82	15.98
	3	37.00	33.44	13.00	10.82	24.48	1.76	16.72
	Average	44.00	45.89	13.67	9.50	21.01	2.45	18.57
HFS + LFSP	1	50.00	33.60	30.00	17.47	36.90	5.60	10.40
	2	50.00	47.88	25.00	9.75	25.62	3.36	14.28
	3	46.00	31.16	24.00	12.26	24.34	1.64	9.84
	Average	48.67	37.55	26.33	13.16	28.95	3.53	11.51
LFS + LFSP	1	51.00	51.15	4.00	14.96	27.03	1.86	19.53
	2	45.00	40.42	10.00	12.99	28.57	2.82	15.98
	3	51.00	40.94	10.00	8.37	15.88	2.58	18.92
	Average	49.00	44.17	8.00	12.11	23.83	2.42	18.14

*HFS + LFSP* High freezability spermatozoa + Low freezability seminal plasma, *LFS + HFSP* Low freezability spermatozoa + High freezability seminal plasma, *FS + HFSP* High freezability spermatozoa + High freezability seminal plasma, *LFS + LFSP* Low freezability spermatozoa + Low freezability seminal plasma

The SPZ × seminal plasma interaction did not affect (*P* > 0.05) any sperm variable; there was seminal plasma effect only on acrosome integrity (*P* < 0.05) and highly significant effect of SPZ (*P* < 0.01) on total motility, acrosome integrity and CS, and significant (*P* < 0.05) on MFI and progressive motility. There was not significant effect (*P* > 0.05) of seminal plasma and SPZ factors on membrane structural integrity and Percentage of functionally competent sperm. When testing for multiple comparisons for acrosome integrity it was observed that a higher value (*P* < 0.05) of 20.80 ± 0.09 with seminal plasma of MHF was obtained compared to seminal plasma found with MLF of 14.51 ± 0.11.

Likewise, in assessing the effect of SPZ on this same variable, combinations with SPZ of MLF showed lower acrosome integrity (*P* < 0.01) than mixtures with SPZ of MHF (10.4 ± 0.12 vs 28.87 ± 0.07). According to the above, there are reports of proteins related to acrosome integrity as the N-acetyl-β-hexosaminidase [4] and proteins of sperm surface, called Aspartic acid-Glutamine-Histidine, DQH [40] with acrosome protective function. In addition, this finding may be related to the action of L-PGDS in the acrosome membrane. In the case of total and progressive motility, in combinations with SPZ of MLF, lower values (*P* < 0.01) total motility (22.37 ± 0.08) were found than those found in combinations with SPZ of MHF ( 31.76 ± 0.07); likewise the progresive motility in SPZ of MLF combinations (10.72 ± 0.12) was lower (*P* < 0.05) than that found with SPZ of MHF (15.05 ± 0.10). There exist reports that associate high intracellular levels of HSP90a with motility [5, 23], and in the present study the SPZ of MLF combinations showed higher amount of this protein in the seminal plasma after 3 h incubation, possibly due to leakage from inside the cell. Other sperm proteins recently associated with freezability and motility are voltage-dependent anion channel 2 which regulate the permeability of the membrane [41], the acrosin binding protein and the triosephosphate isomerase protein connected to the capacitation process [6].

In the analysis of multiple comparisons, the CS values were higher (*P* < 0.01) in the SPZ of MLF (18.35 ± 0.09) compared to the SPZ of MHF (10.39 ± 0.12). This may be related to cholesterol levels in the sperm membrane, leading to a greater or lesser resistance to freezing, or to the difference in the amount of polyunsaturated fatty acids [42], associated to the level of lipid peroxidation by ROS, molecules that can cause cryocapacitation [43].

## Conclusions

The concentration of the HSP90a protein increased in seminal plasma after 3 h of incubation, and this increase was greater in combinations with spermatozoa of low freezability, a fact which is possibly associated with reduction of membrane integrity or degradation of the protein by heat shock.

Two isoforms (16 and 19 kDa) of the NPC2 protein were found in this work. The concentration of 19 kDa was higher in the combinations with seminal plasma of MHF, both at 0 and 3 h of incubation with a lower concentration at the end of incubation, which may be associated with a better prevention mechanism of the cryocapacitation.

The L-PGDS protein levels decreased after 3 h of incubation in the mixtures containing spermatozoa of the male of low freezability, which may be related to the affinity of this protein to retinol and with effect of these on the permeability of the membrane that would lead the start of the cryocapacitation and subsequent acrosome reaction.

The mixtures with low freezability spermatozoa showed greater increase levels of the HSP90a protein and reduction of L-PGDS in seminal plasma after 3 h of incubation; also, the seminal plasma of MHF had higher concentration of the NPC2 of 19 kDa, which was reduced by incubating with MHF spermatozoa. According to the above, it is possible to consider that the three proteins evaluated were associated with freezability of boar semen.

## Abbreviations

BSA: Bovine serum albumin
cAMP: Cyclic adenosine monophosphate
CASA: Computer assisted seminal analysis
CS: Cryocapacitated sperm
HFS + HFSP: High freezability spermatozoa + High freezability seminal plasma
HFS + LFSP: High freezability spermatozoa + Low freezability seminal plasma
HSP: Heat shock proteins
HSP90a: Heat shock protein 90 alpha
IT: Incubation time
LFS + HFSP: Low freezability spermatozoa + High freezability seminal plasma
LFS + LFSP: Low freezability spermatozoa + Low freezability seminal plasma
L-PGDS: Lipocalin-type prostaglandin D synthase
MFI: Membrane functional integrity
MHF: Male with high freezability
MLF: Male with low freezability
MSI: Membrane structural integrity
NPC2: Niemann-Pick disease type C2 protein
PBS: Phosphate buffered saline
PBST: Tween 20 at 0.1% in PBS
PFCS: Percentage of functionally competent sperm
PVDF: Polyvinylidene difluoride
RCU: Relative concentration units
ROS: Reactive oxygen species
SDS-PAGE: Sodium Dodecyl Sulfate Polyacrylamide Gel Electrophoresis
sHOST: Short hypoosmotic swelling test
SPZ: Spermatozoa
